# EHV-1: A Constant Threat to the Horse Industry

**DOI:** 10.3389/fmicb.2019.02668

**Published:** 2019-12-03

**Authors:** Fatai S. Oladunni, David W. Horohov, Thomas M. Chambers

**Affiliations:** ^1^Maxwell H. Gluck Equine Research Center, Department of Veterinary Science, University of Kentucky, Lexington, KY, United States; ^2^Department of Veterinary Microbiology, University of Ilorin, Ilorin, Nigeria

**Keywords:** EHV-1, abortion, myeloencephalopathy, latency, horse

## Abstract

Equine herpesvirus-1 (EHV-1) is one of the most important and prevalent viral pathogens of horses and a major threat to the equine industry throughout most of the world. EHV-1 primarily causes respiratory disease but viral spread to distant organs enables the development of more severe sequelae; abortion and neurologic disease. The virus can also undergo latency during which viral genes are minimally expressed, and reactivate to produce lytic infection at any time. Recently, there has been a trend of increasing numbers of outbreaks of a devastating form of EHV-1, equine herpesviral myeloencephalopathy. This review presents detailed information on EHV-1, from the discovery of the virus to latest developments on treatment and control of the diseases it causes. We also provide updates on recent EHV-1 research with particular emphasis on viral biology which enables pathogenesis in the natural host. The information presented herein will be useful in understanding EHV-1 and formulating policies that would help limit the spread of EHV-1 within horse populations.

## Ehv-1 Infection of Horses: How It All Began

After the discovery of the first virus (Ivanovsky, 1882), 50 years passed before Dimock and Edwards in 1932 first showed that a different kind of microorganism, other than bacteria, was causing contagious epizootic abortion in mares (Dimock and Edwards, 1932). Around the same time, the disease was reproduced by experimental infection of mares with materials from aborted fetuses. Their research indicated that a filterable viral agent was causing abortions in pregnant mares, and they coined the term “viral abortion” to refer to the syndrome (Dimock and Edwards, 1933). They further identified the gross pathological changes in the aborted fetuses, including intranuclear inclusion bodies in fetal pneumocytes and hepatocytes, and established the term ‘equine viral abortions’ to describe the disease (Dimock, 1940). Subsequently, the ‘equine abortion virus’ (EAV) was grown *in vivo* and *in vitro* (Anderson and Goodpasture, 1942; Doll et al., 1953; Randall et al., 1953), and detailed pathological findings were published (Westerfield and Dimock, 1946).

Around the same period, Manninger and Csontos in Hungary also documented the same symptoms of viral abortions as in Kentucky, along with signs of respiratory disease including mild fever (Manninger and Csontos, 1941). They observed the development of symptoms resembling that of mild influenza when bacteriological sterile filtrate from the aborted fetuses with lesions of viral abortion was inoculated into pregnant mares (Manninger and Csontos, 1941). Salyi (1942) also demonstrated that the observed gross and microscopic lesions in fetal abortion material were identical with those reported in Kentucky. In fact, Kress (1941) indicated that the abortion virus is pneumotropic due to the prevalence of bronchopneumonia in horses in contact with aborted materials. This prompted Manninger (1949) to infer that the viral abortion was caused by infection with equine influenza virus in pregnant mares.

Doll et al. (1954b) first studied the respiratory infection associated with EAV, and the symptomatology developed in young inoculated horses was again similar to that described as equine influenza, the cause of which had not yet been identified. The evidence from their research showed that EAV is the etiological agent of epizootic respiratory disease of young horses (Doll et al., 1954b). It remained for Doll and co-workers to show that several putative isolates of the influenza virus were the same as EAV (Doll and Kintner, 1954a; Doll et al., 1954a; Doll and Wallace, 1954b). In another study, Bryans et al. (1957) suggested that the causative agent previously known as EAV should be considered a respiratory virus because of the prominence of the major histological lesions in the respiratory tract of young and aborted foals. The authors, therefore, referred to the virus-induced disease as viral pneumonitis and the agent as an equine viral pneumonitis virus. In 1963, electron microscopy revealed that the virus was a member of the herpes group (Plummer and Waterson, 1963).

## Classification of Herpesviruses

Herpesviruses have undergone significant diversification in terms of virion morphology, biological properties, and antigenic properties (Roizman, 1982). The Herpesviridae family members are classified into three subfamilies: *Alphaherpesvirinae*, *Betaherpesvirinae*, and *Gammaherpesvirinae* (Roizman et al., 1981) based on their morphology and biological properties.

Alphaherpesviruses are found in a wide range of host species. They undergo an efficient and relatively short replicative cycle, and they establish latency in the sensory neurons or lymphocytes of their hosts (Pellet, 2007). They spread well from cell to cell, but are also easily released from infected cells where they replicate, causing cytopathic effects and the development of intranuclear eosinophilic inclusion bodies (Rajcani and Durmanova, 2001). *In vitro*, they are often able to infect cells from different animal species. Although *in vivo* the alphaherpesviruses can infect various host species, there is always a species to which each virus has been adapted (Rajcani and Durmanova, 2001). In such a host, they have the propensity to undergo latency, during which viral pathogenicity is absent. It is suspected that the alphaherpesviruses spread best in the host along the nerves, where intra-axonal transmission predominates (Rajcani and Durmanova, 2001). Members of *Alphaherpesvirinae* subfamily include four different genera; *Simplexvirus*, *Varicellovirus*, *Mardivirus*, and *Iltovirus* (Davison, 2007). EHV-1 is a member of the genus *Varicellovirus*.

Unlike alphaherpesviruses, betaherpesviruses have a limited host range and a long cycle of replication (Riaz et al., 2017). *In vitro*, members of the *Betaherpesvirinae* only replicate in cells derived from their specific host, further underscoring their narrow host range (Rajcani and Durmanova, 2001). They have a slow replication cycle (running for several days), and their release from infected cells is ineffective (Rajcani and Durmanova, 2001). Betaherpesvirus infection slowly progresses in tissue culture and the infected cells become larger rather than lyse and contain intranuclear inclusion bodies (Roizmann et al., 1992; Rajcani and Durmanova, 2001). Latent infection is established predominantly in monocytes or macrophages (Riaz et al., 2017). Since they do not exhibit preferential neural expansion, they typically exist in leukocytes, reticuloendothelial cells, as well as in renal tubular epithelial cells and the salivary gland ducts (Rajcani and Durmanova, 2001). The viruses in this subfamily are further classified into four genera namely *Roseolovirus*, *Proboscivirus*, *Cytomegalovirus*, and *Muromegalovirus* (Davison et al., 2009).

The members of the subfamily *Gammaherpesvirinae* are slow replicating viruses with lymphotropic properties and limited host range (Rajcani and Durmanova, 2001). Unlike alpha- and betaherpesviruses, gammaherpesviruses initially seem to favor the development of latency in either T or B cells, whereas only a subset of cells supports lytic replication (Ackermann, 2006). There are more homolog genes conserved within members of the subfamily *Gammaherpesvirinae* than members of the other two subfamilies (Riaz et al., 2017). In addition to the genes conserved between herpesviruses, each gammaherpesvirus also contains a set of unique genes which are usually present at the terminal regions of the genome and which are important for viral pathogenesis (Riaz et al., 2017). This subfamily consists of four genera: *Percavirus, Macavirus, Rhadinovirus*, and *Lymphocryptovirus* (Davison et al., 2009).

## Equine Herpesviruses

To date, all the nine equid herpesviruses isolated belong to either the *alphaherpesvirinae* or *gammaherpesvirinae* subfamilies (Table 1). The members of the subfamily of alphaherpesviruses include EHV-1, EHV-3, EHV-4, EHV-6, EHV-8, and EHV-9 (Davison, 2007). The members of the gammaherpesviruses include EHV-2, EHV-5, and EHV-7. Only five of the nine herpesviruses (viz. EHV-1, 2, 3, 4, and 5) can produce disease in horses (Allen et al., 2004). EHV-6 to 8 produce diseases in donkeys and are also known as asinine herpesvirus (AHV, AHV-1 to 3), while EHV-9 or gazelle herpesvirus (GHV) is a pathogen of Thomson’s gazelles (Ostlund, 1993; Crabb and Studdert, 1995; Taniguchi et al., 2000).

**TABLE 1:** Known equine herpesviruses.

**Subfamily of *Herpesviridae***	***Equus* species**				***Gazella thomsoni***
	**Domestic horse (*Equus caballus*)**	**Donkey (*Equus asinus*)**	**Zebra (*Equus grevyi*)**	**Onager (*Equus hemionus onager*)**	
*Alphaherpesvirinae*: (a) Viscerotropic subgroup	Equine herpesvirus 1^a^ (*Equid herpesvirus 1*)^b^ Equine herpesvirus 4 (*Equid herpesvirus 4*)	Asinine herpesvirus 3 (*Equid herpesvirus 8*)	Zebra herpesvirus isolates	Onager herpesvirus isolates	Gazelle herpesvirus 1 (*Equid herpesvirus 9*)
(b) Dermatotropic subgroup	Equine herpesvirus 3 (*Equid herpesvirus 3*)	Asinine herpesvirus 1 (*Equid herpesvirus 6*)			
*Gammaherpesvirinae*	Equine herpesvirus 2 (*Equid herpesvirus 2*) Equine herpesvirus 5 (*Equid herpesvirus 5*)	Asinine herpesvirus 2 (*Equid herpesvirus 7*)			

*Table adapted from Allen et al. (2004). ^*a*^Viruses in the same horizontal row of the table are closely related equid herpesviruses with minor antigenic and genetic variation imposed by natural selection for specific host species. ^*b*^Virus nomenclatures in parentheses are assigned by the Herpesvirus Study Group of the International Committee on Taxonomy and Nomenclature of Viruses (ICTV) (Roizman et al., 1995; Davison et al., 2000). At this time, the study group has not given ICTV designations to zebra or onager herpesviruses (Davison et al., 2000). A neurotropic herpesvirus isolate from captive gazelle has provisionally been designated *Equid herpesvirus 9* (Fukushi et al., 1997; Davison et al., 2000; Taniguchi et al., 2000).*

## Epidemiology and Transmission of Ehv-1

Exposure of horses to either EHV-1 or its close relative EHV-4 occurs very early in life. It has been reported that between 80 to 90% of horses are infected, with either pathogen, by the time they are 2 years of age (Allen, 2002). The great level of antigenic relatedness between EHV-1 and EHV-4 often complicates seroepidemiological findings as a result of a lack of type-specific antibodies and extensive antigenic cross-reactivity that exists in natural infection (Patel and Heldens, 2005). In the early 1990s, evidence became available that the envelope glycoprotein, gG, of EHV-4 elicits a type-specific antibody response, which enabled the differentiation between antibodies present in polyclonal sera from mixed cases of infection involving both EHV-1 and EHV-4 (Crabb et al., 1992). The antigenic determinants in the carboxyl domain of the gG’s of EHV-1 and EHV-4 have been described as useful tools for differentiating between these viruses based on distinct humoral responses that they elicit in their natural hosts (Crabb et al., 1992; Crabb and Studdert, 1993). The annual incidence of EHV-1 is not well defined, as a result of mixed infection with EHV-4 and the ability of both viruses to undergo latency. Latency is an important survival strategy employed by alphaherpesviruses for continuous persistence and dissemination within their natural host population (Whitley and Gnann, 1993). Virus reactivation in an infected host, following latency, could occur at any time to promote a clinical course of the disease and virus shedding.

EHV-1 infection is highly contagious and can easily be acquired by contact with infectious materials, including fomites and aerosols (Lunn et al., 2009). Transmission of the virus to susceptible horses is facilitated by contact with an acutely infected horse or a reactivated virus-shedding horse, or from contact with an aborted fetus or placenta which is rich in infectious virus particles (Allen et al., 2004). Extensive work investigating the transmission cycle of EHV-1 has identified mare and foal populations as important reservoirs enabling virus transmission before and after weanling, with infection in foals occurring within the first 30 days of life (Gilkerson et al., 1999). In a different report, viral shedding was detected in 22 day-old foals even after a widespread vaccination of mares (Foote et al., 2004). Evidence suggests that infected mares, especially those incubating latent EHV-1, serve as a continuous source of viral exposure to foals by horizontal transmission when contact is established between foals and the nursing dam. Broodmares may undergo recrudescence of latent viral infection as a result of stress resulting from pregnancy/parturition, which may expose young foals to EHV-1 infections from mares that are actively shedding the virus (Paillot et al., 2008). Overall, available data suggest a cyclic but mostly quiet epidemiologic pattern of EHV-1 infection with an infected dam serving as a continuous source of infectious virus particles to its foals between breeding seasons.

## Genomic Structure and Gene Functions of Ehv-1

The full genome sequence of EHV-1 has been published (Telford et al., 1992, 1998) making information regarding the genomic organization of the virus available. EHV-1 has a linear dsDNA genome of about 150.2 kbp in size with base composition of about 56.7% G+C content (Roizmann et al., 1992). The entire genome is composed of a long unique region (U_*L*_, 112,870 bp) flanked by a small inverted repeat sequence (TR_*L*_/IR_*L*_, 32 bp), and a short unique region (U_*S*_, 11,861 bp) that is flanked by a large inverted repeat (TR_*S*_/IR_*S*_, 12,714 bp) (Roizmann et al., 1992). The genome contains 80 open reading frames (ORFs) encoding 76 different genes, with four duplicated ORFs present in the terminal repeat sequence (TRS) (Telford et al., 1992; Crabb and Studdert, 1996). The four duplicated ORFs in the EHV-1 genome are ORF 64, 65, 66, and 67 which are present in the sequences flanking the unique short segment (Allen et al., 2004). The inverted repeats allow the short components to give rise to virion populations which exist in two orientations, generating two isomeric DNA molecules (Henry et al., 1981; O’Callaghan et al., 1981, 1983; Whalley et al., 1981; Ruyechan et al., 1982). The gene layout of EHV-1 reveals tightly arranged ORFs with little intervening sequence, the absence of extensive ORF overlap, and few instances of exon splicing (Allen et al., 2004). Generally, this gene arrangement of EHV-1 is similar to other sequenced herpesviruses with the only difference being that EHV-1 encodes five genes (ORF1, 2, 67, 71, and 75) which have no structural homolog when compared to all other herpesviruses sequenced to date (Allen et al., 2004). Some of these genes’ functions remain unknown but have been predicted to exert major influence in the unique biology of EHV-1 enabling them to adapt to the horse as their natural host (Allen et al., 2004). The genomic details of EHV-1 ORFs including the functions of individual genes are listed in Table 2.

**TABLE 2:** EHV-1 gene products and their functions.

**EHV-1 ORF #**	**Start**	**Stop**	**Functional class of HSV homolog (core genes)**	**Gene products and proposed functions**
			**Capsid protein assembly**	
43^†^	82083	83027	UL18	VP23, involved in intercapsomeric formation with VP19c
42^†^	77703	81832	UL19	VP5, major capsid protein
35^†^	67093	65153	UL26	VP24 and VP21 are products of self-cleavage of UL26, serine protease
35.5^†^	66142	65153	UL26.5	VP22a, scaffolding protein
25^†^	47311	46952	UL35^∗^	VP26, capsid protein
22^†^	32916	31519	UL38	VP19c, a component of intercapsomeric complex
			**DNA replication**	
57^†^	102375	105020	UL5	DNA helicase-primase, DNA replication
54^†^	97069	99324	UL8	DNA helicase-primase, DNA replication
31^†^	55453	59082	UL29	ICP8, Single stranded DNA-binding protein
30^†^	55184	51522	UL30	DNA polymerase, DNA replication
18	25696	24479	UL42	Double-stranded DNA binding protein, DNA polymerase subunit
7^†^	10301	7056	UL52	DNA primase, DNA helicase-primase subunit
			**DNA cleavage/packaging**	
56^†^	102391	100130	UL6	Associated with capsids, a subunit of portal complex
44^†^	84320	83148	UL15	DNA terminase activity, involved in DNA packaging
45^†^	84480	86600	UL17	Associated with B and C capsids, DNA encapsidation
36^†^	68975	67212	UL25	Capsid protein, involved in packaging of cleaved viral DNA
32^†^	59243	61570	UL28	ICP18.5, pac motif-specific DNA binding activity, DNA packaging protein
28^†^	48763	50625	UL32	Cytoplasmic/nuclear protein involved in DNA cleavage/packaging
27^†^	48791	48369	UL33	DNA packaging protein involved in capsid assembly
			**Nucleic acid metabolism**	
61^†^	108144	107206	UL2^∗^	Uracil-DNA glycosylase
50^†^	91135	92832	UL12^∗^	Alkaline nuclease, involved in viral DNA processing
21^†^	31276	28904	UL39^∗^	ICP6, ribonucleotide reductase large subunit involved with protein kinase activity
9^†^	12115	11135	UL50^∗^	Deoxyuridine triphosphate
			**Envelope glycoprotein**	
62^†^	108843	108147	UL1	gL, forms complex with gH to direct viral entry, egress and cell-to-cell spread
52^†^	94472	93120	UL10^∗^	gM, involved in viral cell-to-cell spread
39^†^	71192	73738	UL22	gH, forms complex with gL to direct viral entry, egress and cell-to-cell spread
33^†^	61432	64374	UL27	gB, VP7, required for viral entry into a cell, forms a dimer and induces neutralization antibody
			**Others**	
55^†^	100332	99421	UL7^∗^	Associated with intracellular capsids, involved in DNA packaging?
51^†^	92784	93008	UL11^∗^	Myristoylated viral protein involved in efficient capsid envelopment and egress
49^†^	89369	91153	UL13^∗^	UL13 PK, tegument protein with protein kinase activity
48^†^	88947	89900	UL14^∗^	Tegument protein with molecular chaperone function
46^†^	86620	87732	UL16^∗^	Tegument protein, located within the intron of UL15, involved in DNA packaging?
37^†^	69897	69079	UL24^∗^	Non-glycosylated membrane-associated protein, neuropathogenic virulence factor?
29^†^	50618	51598	UL31^∗^	Nuclear matrix binding protein, interacts with UL34
26^†^	48230	47403	UL34^∗^	Associate with inner nuclear membrane, required for nuclear egress
24^†^	36588	46853	UL36	ICP1/2, largest tegument protein, involved in both uncoating and egress
23^†^	33292	36354	UL37	ICP32, Tegument protein with nuclear export signal, involved in egress and virion maturation
NA			UL49.5^∗^	Small membrane-associated protein
8^†^	10300	11037	UL51^∗^	Palmitoylated virion protein, associated with the Golgi
5^†^	5874	4462	UL54	ICP27, regulation of gene expression at the post-transcriptional level
			**Non-core essential genes**	
53	94390	97053	UL9	Replication origin-binding protein
12	13595	14944	UL48	VP16, tegument protein involved in (immediate early) IE gene expression
64	118591 144569	114128 149032	RS1	ICP4, major regulatory protein
72	131583	132791	US6	gD, required for virus entry
			**Non-core accessory genes**	
NA			RL1^∗^	ICP34.5, protein synthesis regulator
63	111985	110387	RL2^∗^	ICP0, promiscuous transactivator with E3 ubiquitin ligase domains involved in gene regulation
60	107116	106478	UL3^∗^	Nuclear phosphoprotein, involved in nucleolar localization
58	105070	105747	UL4^∗^	Nuclear protein, co-localized with UL3 and ICP22
41	76793	77512	UL20^∗^	Virion protein, essential for viral exocytosis
40	76224	74632	UL21^∗^	Tegument protein, associated with microtubules
38	69910	70968	UL23^∗^	ICP36, thymidine kinase (TK) required for nucleotide metabolism
20	28859	27894	UL40^∗^	Ribonucleotide reductase small subunit, involved in nucleotide metabolism
19	26262	27755	UL41^∗^	vhs, virion host shut-off protein, causes non-specific cellular mRNA degradation
17	24234	23029	UL43^∗^	Membrane-associated protein
16	22851	21445	UL44^∗^	gC, VP7.5, involved in cell attachment and adsorption, C3b-binding activity
15	21170	20487	UL45^∗^	Virion protein, type-II membrane protein involved in virus egress
14	18083	20326	UL46^∗^	VP11/12, tegument protein, interacts with UL48 (VP16)
13	15317	17932	UL47^∗^	VP13/14, tegument protein, enhances immediate early gene expression
11	12549	13463	UL49^∗^	VP22, tegument protein with intercellular trafficking activity
6	7042	6011	UL53^∗^	gK, required for efficient viral exocytosis
4	4249	3647	UL55^∗^	Nuclear protein, nuclear matrix-binding protein
NA			UL56^∗^	Type-II membrane protein, associated with intercellular trafficking
65	121368 141792	122249 140911	US1^∗^	ICP22, regulatory protein involved in the expression of late genes
68	126275	125019	US2^∗^	Virion protein, interacts with cytokeratin
69	126411	127559	US3^∗^	US3 PK, has anti-apoptotic activity
70	127681	128916	US4^∗^	gG, involved in viral entry and egress
71	129097	131490	US5^∗^	gJ, protects from Fas-mediated apoptosis
73	132899	134173	US7^∗^	gI, interacts with gE, involved in cell-to-cell spread
74	134406	136058	US8^∗^	gE, forms complex with gI, Fc receptor activity, cell-to-cell spread
NA			US8.5^∗^	Localized in nucleoli of infected cells
76	136783	137442	US9^∗^	Type-II membrane protein, involved in anterograde transport of envelope glycoprotein?
66	122862 140298	123572 139588	US10^∗^	Tegument protein, tightly associated with capsids
NA			US11^∗^	Tegument protein, RNA-binding activity, and intercellular trafficking
NA			US12^∗^	TAP-binding protein, involved in MHC class I downregulation
1	1298	1906	NA	Downregulates MHC class I
2	2562	1945	NA	Virion virulence factor
3	2841	3614	NA	Unknown
34	64578	65060	NA	Unknown
47^†^	88917	87886	NA	Unknown
10^†^	12084	12386	UL49A	gN, Envelope protein
59	106416	105877	NA	V57, virion morphogenesis?
67	125194 137966	124376 138784	NA	VP67, co-localizes with nuclear lamin
75	136055	136447	NA	US8A, unknown function

*Table was prepared according to available data published in [opetwcite]B5,B172[clotwcite]Allen et al. (2004); Nishiyama (2004), Davison (2010), Sarkar (2014), [opetwcite]B213,B122[clotwcite]Roizman (1996); Kasem et al. (2010), Telford et al. (1998), and Ma et al. (2012). ^∗^Indicates that the gene is dispensable for replication in cell culture; NA, not available; ^†^indicates conserved genes across all mammalian herpesviruses.*

## Biological Functions of Ehv-1 Proteins

**FIGURE 1:**
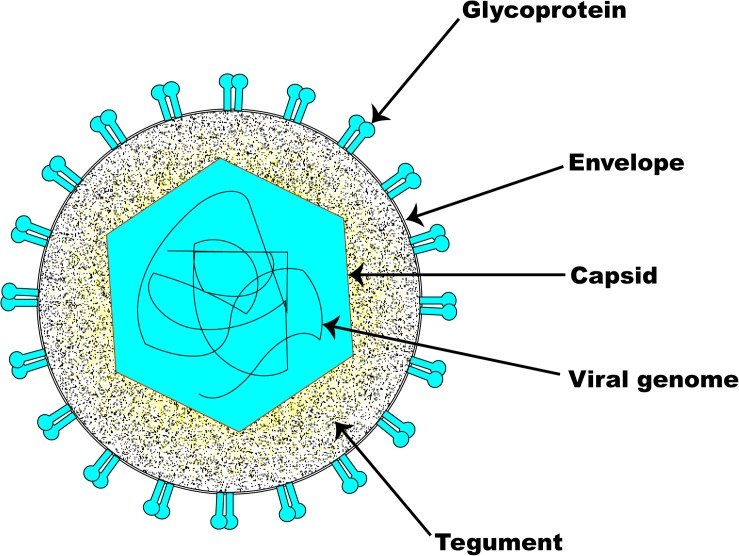
Schematic illustration of EHV-1 structure. The basic architecture of EHV-1 consists of an envelope, a tightly woven dsDNA genome enclosed within the capsid, and a tegument layer. Several glycoproteins are present on the surface of the envelope.

The structural architecture of a typical EHV-1 particle (Figure 1) is made up of about 30 discrete kinds of polypeptides (Perdue et al., 1974; Turtinen et al., 1981; Turtinen and Allen, 1982; Turtinen, 1984; Meredith et al., 1989). It consists of a genomic core made up of a linear double-stranded DNA neatly packed within an icosahedral capsid of T = 16 with an approximate diameter of 100–110 nm (Riaz et al., 2017). The nucleocapsid, which houses the viral genome, is in itself made up of six proteins encoded by ORFs 22, 25, 35, 42, 43, and 56 (Perdue et al., 1974; Allen et al., 2004). The capsids of all herpesviruses are similar, comprising 162 capsomers (12 pentons and 150 hexons) (Paillot et al., 2008). The nucleocapsid contains a ring structure made up of 12 portal proteins which enables viral DNA to enter into the capsid (Newcomb et al., 1989; Baker et al., 1990). Although their names differ between herpesvirus families, capsid protein structure and arrangement are preserved across all herpesviruses (Baines, 2011; Brown and Newcomb, 2011).

The amorphous tegument layer, which corresponds to the area between the nucleocapsid and the envelope, comprises about twelve different proteins encoded by ORFs 11, 12, 13, 14, 15, 23, 24, 40, 46, 49, 51, and 76 (Allen et al., 2004). These tegument proteins and enzymes are critically involved in very early events during infection which are required for initiating viral replication (Batterson et al., 1983; Coulter et al., 1993; Paillot et al., 2008). The large tegument protein, UL36, interacts with the capsid’s pentons (VP5), which gives an icosahedral symmetry to the innermost portion of the tegument (Machtiger et al., 1980; Newcomb et al., 1996; Zhou et al., 1999). The outermost part of the tegument engages with the envelope membrane of the virus and may sometimes associate with the intravirion part of the integral membrane proteins.

Surrounding the nucleocapsid and the tegument is the viral envelope derived from patches of an altered host-derived cell membrane (Riaz et al., 2017). Embedded in the EHV-1 envelope are eleven glycoproteins which are functional homologs of those found in HSV-1. The eleven EHV-1 glycoproteins (i.e., gB-gp14, gC-gp13, gD-gp18, gE, gG, gH, gI, gK, gL, gM, and gN) are preserved across all alphaherpesviruses and are therefore named according to the HSV-1 nomenclature (Paillot et al., 2008). As with other herpesviruses, the envelope glycoproteins of EHV-1 are critical determinants of virus entry into a susceptible host cell, host range, virus cell-to-cell spread, pathogenicity, and immunologic responses to infection. EHV-1 encodes an extra gp2 that has homologs only in AHV-3 and EHV-4 (Paillot et al., 2008). The inclusion of tegument and viral envelope enables the virion size to markedly increase from 120 nm to approximately 300 nm (Roizmann et al., 1992).

## Cell Infection and Virus Replication

**FIGURE 2:**
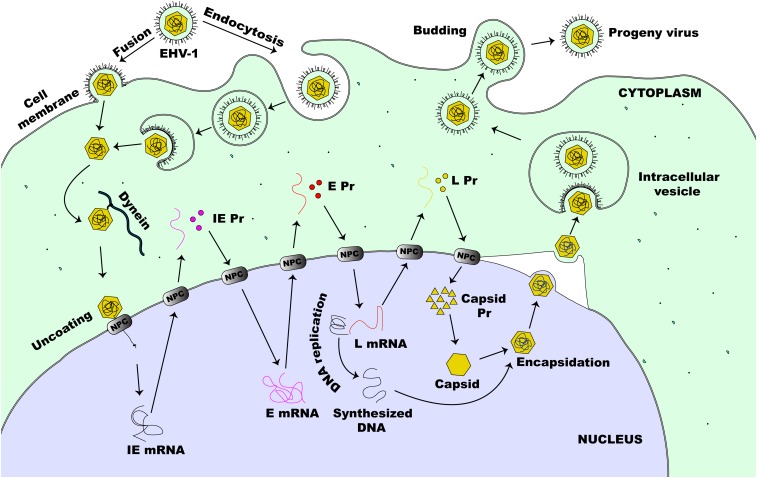
Lytic life cycle of EHV-1. The virus enters susceptible cells either by fusion at the cell membrane or by the non-classical endocytosis pathway. This is followed by the release of nucleocapsid into the cytoplasm of an infected cell. The nucleocapsid, which is transported to the nucleus via dynein, docks at the NPC and extrudes the viral DNA directly into the nucleus. This initiates viral gene expression beginning with the transcription of IE (α) gene. Immediate early proteins are then synthesized in the cytoplasm and migrate to the nucleus where they direct the transcription of E (β) genes. Early proteins, synthesized in the cytoplasm, translocate to the nucleus to initiate virus DNA replication and virus L (γ) gene expression. Next, some of the L proteins synthesized in the cytoplasm, migrate to the nucleus to form the capsid before encapsidation of the new virus DNA. The newly assembled virion then migrates through the nucleus and the cytoplasmic membranes before it is eventually released outside of the cell.

The lytic replication cycle of EHV-1 can be summarized as follows: entry into a permissive host cell, viral nucleocapsid uncoating, viral gene expression, viral DNA replication, virion assembly, and virion particle egress (Figure 2). In horses, EHV-1 can infect a diverse range of cell types, including endothelial cells of inner organs, epithelial cells of the respiratory tract, and mononuclear cells in lymphoid organs and the peripheral blood (PBMCs) (Osterrieder and Van de Walle, 2010). Cells are either infected by direct contact with an infectious EHV-1 particle or by cell-to-cell spread following contact with an infected cell (Paillot et al., 2008). Like HSV-1 and most other alphaherpesviruses, including EHV-1, efficient infection is initiated by a relatively unstable attachment to heparan sulfate molecules on the proteoglycan cell surface, mediated by gC and gB, followed by binding of gD to one of the specific receptors on the cell surface (Osterrieder, 1999; Spear, 2004; Azab et al., 2010). The changes in conformation as a result of receptor engagement with gD enables intricate interactions between gB and gH/gL (Spear, 2004). However, for virus entry into host cells, EHV-1 also utilizes a unique receptor that is different from those described for other alphaherpesviruses (Frampton et al., 2005). It has been shown that the Major Histocompatibility Complex I (MHC-I) molecules on some equine cells serve as entry receptors for EHV-1 by binding directly to gD on the viral envelope membrane (Kurtz et al., 2010; Sasaki et al., 2011).

EHV-1 can enter permissive cells either by direct fusion with the host cell membrane or by cell-mediated endocytosis, producing a productive infection in both cases (Frampton et al., 2007). Both entry pathways facilitate the release of viral nucleocapsid and tegument proteins into the infected host cell. As with other alphaherpesviruses, once the virus is released inside the host cell, the tegument proteins dissociate from the nucleocapsid and the capsid is transported along microtubules via dynein, a minus-end-director motor protein, to the nucleus of the cell (Paillot et al., 2008). This mechanism of capsid transport is important especially in the infection of cells such as neurons where the virus may have to travel a long distance away from the site of infection to reach the nucleus (Paillot et al., 2008; Kukhanova et al., 2014). Following the arrival of the capsid at the nucleus, the capsid directly binds to the nuclear pore complex (NPC) and extrudes its content into the nucleus leaving the capsid behind in the cytoplasm (Whittaker and Helenius, 1998; Ojala et al., 2000). For HSV-1, the inner tegument protein UL36 (ICP1/2) which bears a nuclear localization signal (Ojala et al., 2000), together with nucleoporins Nup358 and Nup214 which both bind either directly or indirectly to the capsid, facilitate this process (Kukhanova et al., 2014). Seemingly, all these associations and interactions are necessary for the nuclear import of the viral DNA by importin β (Copeland et al., 2009).

Once in the nucleus, the virus can transcribe and replicate its genome, which is a critical step toward virus progeny assembly (Kukhanova et al., 2014). These events lead to the reorganization of the nucleus resulting in increased nucleus size, nucleolus and nuclear domain-10 (ND-10) disruption, chromatin condensation and eventual degradation along with nuclear lamina destruction in late infection (Everett et al., 1998; Simpson-Holley et al., 2005; Callé et al., 2008). Six regulatory proteins expressed as one IE protein (IEP), four early proteins (EICP0, EICP22, EICP27, and IR2) and the late EHV-1 α-gene trans-inducing factor (ETIF or VP16) control the coordinated transcription of EHV-1 genes (Caughman et al., 1985; Gray et al., 1987a; Bowles et al., 1997, 2000; Buczynski et al., 1999; Derbigny et al., 2002). This cascade starts with the tegument VP16 (HSV) homolog protein of EHV-1 acting as a transactivator of an IE (α) gene expression (Purewal et al., 1994). During viral entry, VP16 is carried into the infected cell as a tegument protein and is required for efficient initiation of the lytic replicative cycle of the virus (Lewis et al., 1997; Paillot et al., 2008). The IEP of EHV-1 is encoded by ORF64 and synthesized by host cell RNA polymerase II (Gray et al., 1987a, b). The IEP, a polypeptide of 1487-amino acids (aa), is encoded in each of the two inverted repeats (Grundy et al., 1989) and is essential for virus replication (Garko-Buczynski et al., 1998). It stimulates the expression of heterologous viral promoters during the initial stages of infection, self-regulates its own expression, and acts synergistically with EICP22 and EICP27 to activate the expression of early (E or β) and late (L or γ) viral genes (Smith R.H. et al., 1992; Matsumura et al., 1993; Holden et al., 1995; Zhao et al., 1995). For the transcription of IE genes of HSV-1, the cellular transcription factor Oct-1 binds to a unique canonical sequence: 5′-GyATGnTAATGArATTCyTTGnGGG-3′ (where r is a purine base, y is a pyrimidine base, and n is any base) that overlaps the transcription initiation site of the IE promoter (Mackem and Roizman, 1982; Kukhanova et al., 2014). The VP16 protein then interacts with Oct-1 and forms a complex along with the HCFC1 protein which then activates IE gene transcription (Kukhanova et al., 2014). As with HSV-1, EHV-1 also encodes a similar consensus sequence within its IE promoter region (Purewal et al., 1992), however, other octamers within EHV-1 IE promoter seem to participate in ETIF/Oct-1 complex formation which directs the transactivation of EHV-1 IE genes (Elliott and O’hare, 1995).

The E genes of EHV-1, encoding additional regulatory proteins (EICP27, EICP22, and EICP0) along with proteins involved in viral genome replication, are then transcribed (Caughman et al., 1985; Holden et al., 1992, 1995; Zhao et al., 1992; Bowles et al., 1997). Transcription of the EHV-1 E genes occurs before viral DNA synthesis is initiated and is tightly regulated by IEP (Bowles et al., 2000). The IR2 gene is located in the IE gene and encodes an early protein that is a shortened version (aa 323–1,487) of the IEP (Harty and O’Callaghan, 1991). While IR2 protein can trans-repress the IE gene expression, E and L gene expression cannot be trans-activated due to the lack of an IEP *trans*-activation domain encoded by amino acid residues 3–89 (Smith et al., 1994). An early nuclear phosphoprotein, about 419 bp, encoded by the EICP0 gene, can trans-activate all types of EHV-1 promoters (Bowles et al., 1997, 2000). Toward its N terminus, the EICP0 contains a conserved cysteine-rich zinc RING finger domain (C3HC4 type) that is important for the activation of the promoters of E (β) and L (γ1 and γ2) genes (Bowles et al., 2000). Although both the IE and the EICP0 proteins of EHV-1 can *trans*-activate the promoters of EHV-1, their relationship is antagonistic rather than being synergistic (Kim et al., 1999; Bowles et al., 2000). The E genes of EHV-1 encode proteins that are critical for its replication, while the L genes encode the viral structural proteins (Paillot et al., 2008). Following the model of HSV-1 replication, it is now known that once the E proteins are synthesized, viral DNA replication will be started. This involves the interplay of at least seven early proteins including the gene products of UL5, UL8, UL9, UL29, UL30, UL42, and UL52 (Rixon et al., 1996; Davison, 2010; Muylaert et al., 2011). The initial step of replication of HSV DNA involves the separation of the double-stranded helical structure by ICP8 (UL29) or UL9 proteins in the AT-rich domains of the oriL or oriS origin of replication (Kukhanova et al., 2014). The latter has a copy in the U_*L*_ region and two copies in the U_*S*_ region of the herpesviral genome respectively (Kukhanova et al., 2014). ICP8 and UL9 bind specifically to ssDNA fragments and the oriS, respectively. This interaction enables the unwinding of the double-stranded helical structure, which then allows for the loading of the helicase-primase complex (UL52, UL8, and UL5) (Kukhanova et al., 2014). Following unwinding of the dsDNA, a complex of processivity factor UL42 and viral DNA polymerase (UL30) synthesize the leading and the lagging strand of the DNA (Kukhanova et al., 2014). This replication occurs in a rolling circle form termed ‘theta form of replication,’ the mechanism of which has not yet been identified. Such replication ensures the formation of long head-to-tail viral DNA concatemers, which are then cut into separate units when viral DNA is packaged into capsids (Roizman and Knipe, 2001).

In addition to the viral factors, other cellular components involved in the replication of the viral genome include DNA ligase, topoisomerase II, and various DNA repair and homologous recombination systems (Weller and Coen, 2012). Another important factor for viral DNA replication is cellular chaperone protein Hsp90 which is essential for intranuclear localization of viral DNA polymerase (Burch and Weller, 2005). Some viral proteins such as uracil *N*-glycosylase (UL2), deoxyuridine triphosphatase (UL50), thymidine kinase (UL23), alkaline nuclease (UL12), and ribonucleotide reductase (UL39, UL40) participate in nucleotide metabolism, viral DNA synthesis, and DNA repair (Kukhanova et al., 2014).

The production of late (γ) viral genes peaks only after replication of viral DNA has commenced and requires ICP27, ICP8, and ICP4 for an optimal transcription efficiency (Rybachuk, 2009). γ1 (leaky-late) genes such as ICP5 (major capsid protein), ICP34.5, gD, and gB are expressed throughout infection, increasing in transcription only a few fold after replication of DNA has commenced while expression of γ2 (true-late) genes such as UL41 (VHS), UL38, UL36, UL20, gC, and gK, does not occur in significant amounts until after replication of DNA (Rybachuk, 2009). The increase in the expression levels of the late genes, especially those encoding for viral capsids, just after DNA replication has been initiated enables the assembly of progeny virion particles (Kukhanova et al., 2014).

**FIGURE 3:**
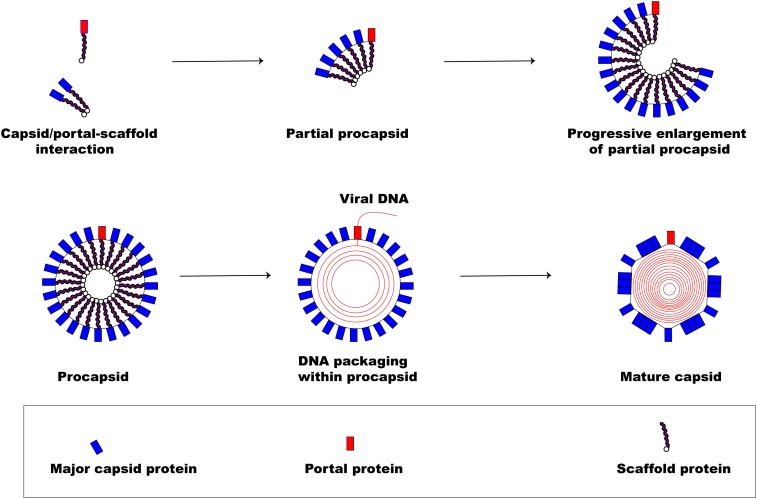
Herpesvirus capsid formation. The basic assembly of a matured capsid consists of complex formation between major capsid protein and scaffold protein with the incorporation of a portal protein. An early step in the formation of a spherical procapsid (partial procapsids) involves complex interactions between the major capsid protein and the scaffold protein to produce what, first, looks like an angular segment. A complex of portal and scaffold protein is incorporated with the progressive enlargement of the partial procapsid. Once the procapsid is formed, virus DNA is packaged marking the exit of the scaffolding proteins creating a polyhedral shape.

Figure 3 illustrates the pathway involved in herpesviral capsid formation. The assembly of the herpesviral nucleocapsid occurs in the nucleus first as a DNA-free precursor capsid in the presence of scaffolding proteins just before viral DNA encapsidation (Perdue et al., 1976; Lee et al., 1988; Rixon et al., 1988; Paillot et al., 2008). The first step in the capsid formation involves the auto-catalytic assembly of a procapsid following the association of pUL6 and pUL19 with a scaffold made up of conserved pUL26 and pUL26.5 proteins (Mettenleiter et al., 2006). The interaction of these proteins produces an angular portion of the spherical procapsid enhanced by scaffold-scaffold complex formation and triplexes connected to VP5 molecules (Brown and Newcomb, 2011). This is then followed by progressive enlargement of the angular segments, called partial procapsids, to form an enclosed spherical procapsid (Newcomb et al., 1996). Although the procapsid appears to be spherical rather than being polyhedral, it has similar diameter (125 nm) and symmetry (T = 16) as the mature capsid (Brown and Newcomb, 2011). In a similar fashion as the major capsid protein VP5, the portal is incorporated into the developing procapsid by interacting with scaffold proteins to form a complex (Newcomb et al., 2003; Singer et al., 2005; Huffman et al., 2008; Yang and Baines, 2008).

Following the formation of the procapsid, the viral dsDNA genome is then packaged into the capsid (encapsidation) regulated by a three subunit protein known as terminase (Yang et al., 2007). The transport of the virion genome into the capsid marks the exit of the scaffold proteins from the procapsid creating the polyhedral shape of the mature capsid.

**FIGURE 4:**
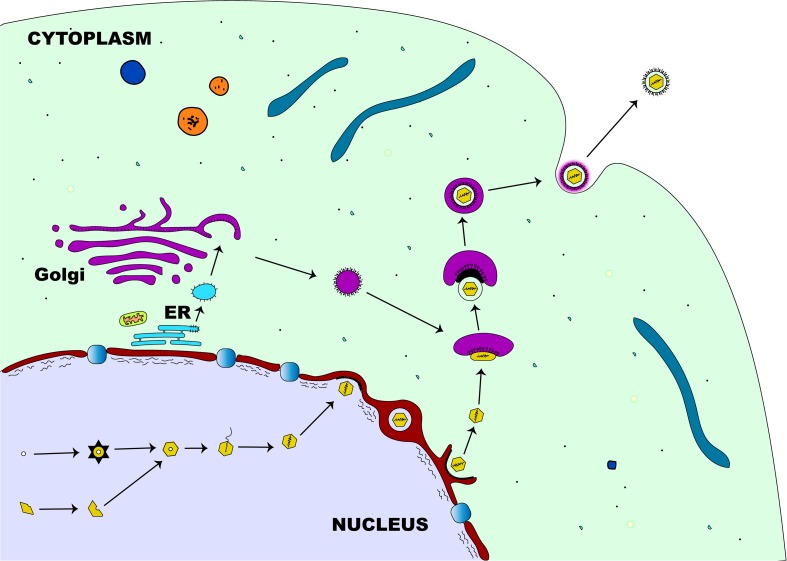
Herpesvirus egress pathway. Following intranuclear encapsidation of the virus genome, the herpesviral nucleocapsid will bud through the inner nuclear membrane resulting in perinuclear localization of an enveloped primary virion. This primary envelope becomes lost (de-envelopment) as the virus translocates into the cytosol where the nucleocapsid acquires tegument proteins. Final (secondary) envelopment then occurs in the cytoplasm derived from the *trans*-Golgi network and the enveloped virion is transported in a vesicle to the plasma membrane for release.

The first step in the egress of herpesviruses from the nucleus is the budding phase. During this stage, the capsid, which is surrounded by tegument proteins, acquires an envelope from the nuclear membrane’s inner leaflet (Mettenleiter, 2002). After the viral genome has been packaged and assembled, the nucleocapsid travels within the nucleus with the aid of actin filament (Forest et al., 2005) to establish contact with the inner membrane of the nucleus before primary envelopment (Figure 4). Two viral proteins (pUL31 and pUL34) that are structurally and functionally conserved across herpesviruses (Mettenleiter et al., 2006) are required for the partial dissolution of the nuclear lamina enabling the nucleocapsid to interact with the inner leaflet of the nuclear membrane (Reynolds et al., 2004). Complex formation between these two proteins is essential for the process of primary envelopment, and the lack of either protein stalls the process of nuclear egress tremendously (Chang et al., 1997; Klupp et al., 2000; Roller et al., 2000; Reynolds et al., 2001; Fuchs et al., 2002b). The association of the pUL31-pUL34 complex with nuclear lamins A/C or B (Reynolds et al., 2004; Gonnella et al., 2005) leads to the recruitment of cellular protein kinase C (PKC) which then activates intranuclear lamins A/C and/or B (Muranyi et al., 2002). This complex interaction leads to the breakdown of the nuclear lamin network and the underlying structures (Reynolds et al., 2004; Simpson-Holley et al., 2005), enabling the nucleocapsid to interact with the inner nuclear membrane (Mettenleiter et al., 2006). Although the nucleocapsid acquires its primary envelope through the process of budding from the inner nuclear membrane, there is a striking difference in morphology and protein content when compared to the mature virus (Granzow et al., 2001; Miranda-Saksena et al., 2002). While the primary envelope contains both pUL31 and pUL34 proteins (Fuchs et al., 2002b; Reynolds et al., 2004), the mature virus particle lacks these two proteins demonstrating the differences in composition between primary and matured virions (Mettenleiter et al., 2006). The underlying mechanism by which the enveloped nucleocapsid gain access into the cytoplasm is not well understood. However, it has been shown that entry into the cytoplasm is by direct fusion of the enveloped nucleocapsid with the outer nuclear membrane (Mettenleiter et al., 2006) rather than exit through the nuclear pore. This eventually leads to loss of envelope (de-envelopment) enabling the naked nucleocapsid to acquire tegument proteins once inside the cytoplasm (Mettenleiter, 2002). The phosphorylation-mediated activation of a portion of primary-enveloped virions achieved by the kinase activity of pUS3, a component of these particles in itself, is required for a successful de-envelopment process (Klupp et al., 2001; Reynolds et al., 2002; Schumacher et al., 2005).

Final tegumentation and secondary envelopment occur in the cytoplasmic compartments and require a highly coordinated network of protein-protein interactions (Mettenleiter et al., 2006). The herpesviral proteins interact on one side with the capsid and on the other side with the cytoplasmic tails of the envelope glycoproteins, enabling the structural integrity of the matured virus particle (Mettenleiter, 2002). Two subassemblies, the capsid and the future envelope, are distinct sites where final tegumentation takes place and they efficiently combine to produce the mature virion (Mettenleiter et al., 2006). The capsid proximal proteins consist of conserved pUL36 and pUL37 that contribute to the physical structure of the tegument, the conserved pUL25, and pUS3 which remains closely associated with the capsid (Mettenleiter et al., 2006). Except for pUS3, the other known components of the inner tegument are preserved across all herpesviruses. Both pUL36 and pUL37 remain closely linked to the capsid until they reach the nuclear pore (Granzow et al., 2005; Luxton et al., 2005) and also serve as a vehicle for intracytoplasmic transport of the capsid during entry and exit of the cytoplasm (Luxton et al., 2006; Wolfstein et al., 2006). Apart from these conserved components, other non-conserved proteins may associate with the inner tegument (Mettenleiter et al., 2006). Strikingly, the abundance of both pUL36 and pUL37 inner tegument proteins in the virion is tightly controlled, unlike those of the outer tegument which vary widely (Michael et al., 2006). Once the outer tegument is added, the virion acquires its secondary envelope. This process of final envelopment occurs within the trans-Golgi network, where glycoproteins together with a subset of tegument proteins (Turcotte et al., 2005), like pUL46, pUL47, and pUL49 for the alphaherpesviruses, are incorporated (Mettenleiter et al., 2006). Two conserved proteins, glycoprotein M and pUL11, that are important for this process have been identified. Glycoprotein M, an envelope protein of the matured virion, helps in retrieving envelope glycoproteins from the cell surface and retaining them at the envelopment site (Crump et al., 2004), while pUL11, a small myristoylated protein, directs envelope protein to future envelope sites (Bowzard et al., 2000; Loomis et al., 2003). At this stage, complex associations between many different proteins are required for the ultimate assembly of a mature herpes virion (Fuchs et al., 2002a; Chi et al., 2005; Mettenleiter, 2006). Following the development of a secondary envelope, a mature herpesviral particle enclosed within an intracellular vesicle is formed and transported to the cell membrane (Mettenleiter et al., 2006), by anterograde cellular microtubule-dependent molecular motor kinesin (Guo et al., 2010). The fusion between this intracellular vesicle and cell membrane enables the release of the newly produced virion particles outside of the cell (Mettenleiter et al., 2009).

## Establishment of Latency

EHV-1, like other herpesviruses, can establish a lifelong presence within cells of a susceptible host following primary infection. The initial stages of EHV-1 infection of the epithelial upper respiratory tract (URT) are accompanied by progression into a stage of latency in which infected horses show no clinical signs of the disease, virus shedding, or cell-associated viremia (Allen et al., 2004; Paillot et al., 2008). While productive infection by EHV-1 leads to active viral gene expression in a well-coordinated manner as described above, the hallmark of latency is the restriction of viral gene expression which culminates in failure to synthesize viral factors and absence of infectious virus particles. The primary site of latency establishment by EHV-1 in the horse has been a subject of debate. While some studies have demonstrated that latency of EHV-1 occurs in lymphocytes, both circulating and those in draining lymph nodes (Welch et al., 1992; Edington et al., 1994; Chesters et al., 1997), others have shown that the sensory nerve cell bodies within the trigeminal ganglia are the preferred primary site of latency for EHV-1 (Slater et al., 1994b; Baxi et al., 1995). While about 80% of CD5+/CD8+ T- lymphocytes have been demonstrated as the major lymphoid cell population enabling latency of EHV-1, a smaller sub-population of 20% CD5+/CD8−/CD4− cells have also been found to support latency (Smith et al., 1998). Regardless of the site of establishment, it appears that the ability of EHV-1 to pass into a latency stage is a deliberate biological behavior that the virus utilizes to perpetuate itself in the host and this enables viral spread to susceptible horses upon reactivation. During latency, the expression of the EHV-1 genome is suppressed and only the latency-associated transcripts (LATs) antisense to either the immediate-early viral gene (ORF 64) or a regulatory early gene (ORF 63) are present in infected cells (Baxi et al., 1995; Chesters et al., 1997; Paillot et al., 2008). The exact molecular and physiological mechanisms that direct latency in EHV-1 infected horses are poorly understood. However, latency has been much better studied in HSV-1 and findings reveal that the major detectable transcript lies within an 8.6-kb sequence antisense to and overlapping the immediate-early (IE) gene IE-1 (ICP0) (Chesters et al., 1997). The LAT itself is a 2.0 kb transcript lacking a polyadenylation site and found mostly in the nuclei of infected neurons (Chesters et al., 1997). In HSV, LAT can promote latency but is dispensable for maintenance or viral reactivation from latency (Efstathiou and Preston, 2005).

It has been shown that reactivation of latent EHV-1 is possible following exposure to stressful conditions such as transportation, handling, re-housing, and weaning or following the administration of corticosteroids (Burrows and Goodridge, 1984; Edington et al., 1985; Slater et al., 1994a). The fact that EHV-1 has been experimentally reactivated from cases of both natural and experimental infection following administration of immunosuppressant (Edington et al., 1985, 1994; Slater et al., 1994b) suggests that horses harboring latent EHV-1 could periodically shed the virus following exposure to stressors. Viral factors, such as the thymidine kinase, have been shown to modulate EHV-1 virulence and latency (Xie et al., 2019).

As a result, the cycle of persistent latent infection followed by reactivation of the virus with shedding into nasal mucus may enable virus propagation and disease spread to susceptible uninfected horses. In certain instances, the characteristic respiratory illness followed by nasal shedding is absent following EHV-1 reactivation and such horses are therefore silent virus shedders (Edington et al., 1985). It has been reported that during the reactivation process a small fraction of lymphocytes carrying the latent EHV-1 genome can progress toward active transcription resulting in DNA revival and fusogenic viral glycoprotein expression on their cell surfaces ultimately leading to active virus replication (Edington et al., 1985; Slater et al., 1994a). The fine details of the molecular mechanism underlying reactivation of EHV-1 from its quiescent state to a lytic productive infection remain elusive. However, it has been suggested that the IE gene promoter of a latent EHV-1 can be trans-activated by the presence of another equine herpesvirus, EHV-2, in a mixed infection (Purewal et al., 1992).

## The Economic Importance of Ehv-1 to the Us Horse Industry

About 10 million of the world horse population reside in the United States (Smith R.H. et al., 1992. FAOSTAT: Livestock 2017). The horse industry is a large and economically diverse industry which accommodates a wide array of economic activities. It has been reported that in the US, the horse industry generates annually an income of about $102 billion when considering both direct and indirect spending (Anonymous, 2005). With such enormous revenue generated from the horse industry in the US, an outbreak of any disease affecting its horse population is likely to perturb the economic health of the industry. The relevant effects of EHV-1 on the equine industry have been summarized (Lunn et al., 2009). Firstly, EHV-1 outbreaks may result in cases of subclinical to mild respiratory illness especially with young athletic horses developing pyrexia and thus lead to interruptions of training schedules. This can be considered as the least significant economic effect of EHV-1 on the horse industry. Secondly, the incidence of abortion in pregnant mares during the third trimester of gestation results in major losses to the growth of the industry. Thirdly, neurologic outbreaks of the disease, equine herpesviral myeloencephalopathy (EHM), are very severe and may lead to deaths of horses, disruption of breeding or training schedules, cancelation of horse events, and extensive movement restrictions with consequent management difficulties at racetracks, training facilities, and other horse shows. Even though horses may recover from the disease, their productivity is usually compromised, and the money expended in the care and management of horses infected with EHV-1 may run into several thousands of dollars depending on the farm size.

## Pathogenesis and Disease Manifestations

The pathogenesis of EHV-1 infection has been described by the study of an experimental model of infection using the EHV-1 strain, AB4 (Paillot et al., 2008). EHV-1 is a highly contagious viral pathogen of horses usually transmitted following direct contact with infectious materials such as nasal discharges and materials from aborted fetuses or indirectly by fomites (Allen et al., 2004). In horses lacking protective mucosal immunity, nasal and mucosal epithelial cells are the primary sites of replication of EHV-1 (Patel et al., 1982; Kydd et al., 1994a; Rusli et al., 2014). Subsequently, virus replication is quickly followed by erosions of epithelial cells of the URT due to necrosis and inflammatory cellular responses which ultimately lead to nasal shedding of infectious virus (Paillot et al., 2008). Once in the URT, EHV-1 can spread quickly utilizing and hijacking infected mucosal monocytes to invade the deeper connective tissues (Gryspeerdt et al., 2010; Vandekerckhove et al., 2010). Consequently, EHV-1 can cross the basement membrane, invading the reticuloendothelial system and the lymphatics to infect circulating leucocytes and endothelial cells of blood vessels (Gryspeerdt et al., 2010). Within 24 h of infection, infected mononuclear leucocytes could be found present in the sinuses and parenchyma of respiratory tract-associated lymphoid organs (Kydd et al., 1994a). Here, EHV-1 undergoes a second round of replication and viral particles are significantly amplified culminating in infected leucocytes escaping, via the efferent lymph, into the blood-vascular circulation leading to a state of cell-associated viremia (Patel et al., 1982; Dutta and Myrup, 1983; Scott et al., 1983; Edington et al., 1986). The ability to establish viremia is key and defines the outcome of EHV-1 pathogenesis produced from the second round of replication. Viremia facilitates the spread of the virus to tertiary replication sites in the endothelium of the pregnant uterus or the central nervous system (Allen et al., 2004) leading to two clinically important sequelae of EHV-1 respiratory infection, namely abortion or a neurological syndrome (Mumford et al., 1994; Slater et al., 1994a).

### Respiratory Disease

**FIGURE 5:**
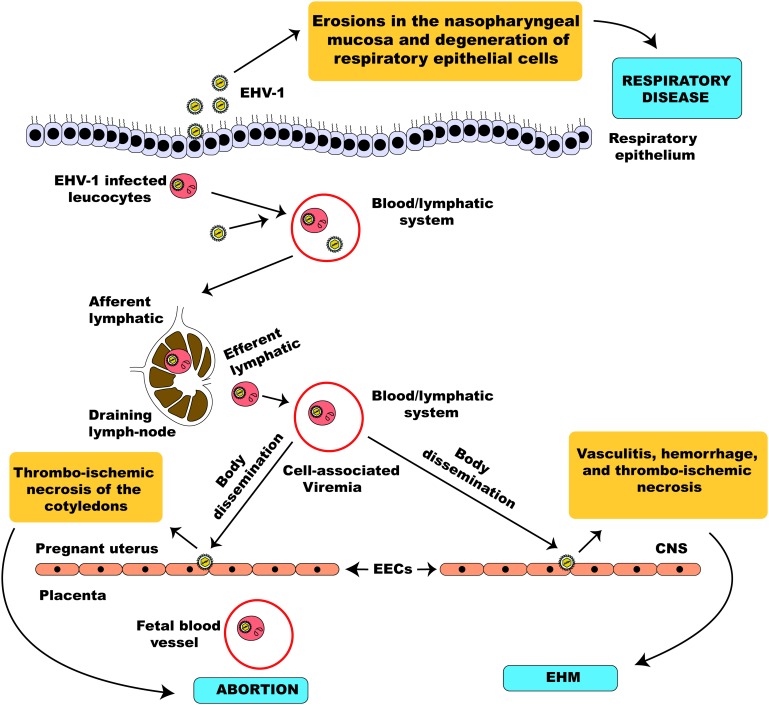
Schematic illustration of EHV-1 pathogenesis. EHV-1 primarily infects the respiratory epithelial cells. The virus can cross the basement membrane and invade the lamina propria where it infects circulating leucocytes. The virus then travels through the lymphatic system to regional lymph nodes where it undergoes amplification and infects peripheral blood mononuclear cells leading to a state of cell-associated viremia. The cell-associated viremia ensures that EHV-1 is disseminated to distant sites such as the endometrium of the pregnant uterus and the CNS causing inflammatory responses that culminate in the development of pathologies; abortion and EHM.

EHV-1 is a leading etiological agent of respiratory disease in horses, producing upper airway infection primarily in young horses. The virus is highly ubiquitous among horse populations causing an epidemic disease in young horses and having an estimated prevalence rate of 80 – 90% in horses below 2 years of age (Allen, 2002). Following contact with an infectious viral particle, the mucosal epithelial cells of the URT of an infected horse are the prime target of EHV-1 where the virus undergoes its first round of replication (Figure 5). Within 12 h post-infection, progeny virus and viral antigen are detectable in the respiratory epithelium of an infected horse (Paillot et al., 2008) and the virus can quickly spread to the respiratory endothelium within 24 h of infection (Kydd et al., 1994b). Besides, circulating mononuclear cells and endothelial cells of blood vessels are also infected as a result of viral cell-to-cell spread, thus facilitating viral dissemination throughout the body (Paillot et al., 2008; Vandekerckhove et al., 2011).

Subsequently, there is erosion and necrosis of the respiratory epithelium, release of proinflammatory cytokines, and shedding of infectious virus particles within the first week of the respiratory disease (Paillot et al., 2008). Depending on the pathogenicity of the EHV-1 strain, the incubation period of infection may either be short (1–3 days) (Gibson et al., 1992a, b,c) or prolonged (up to 10 days) (Ostlund, 1993). EHV-1 primarily results in respiratory tract disease (rhino-pharyngitis and tracheo-bronchitis) (Allen, 2002) presenting a clinical picture similar to other viral pathogens of the respiratory tract of the horse (e.g., equine arteritis virus, influenza virus, adenovirus, or rhinovirus) (Allen, 2002). Although most of these respiratory infections are subclinical or mild, and a large number of foals seroconvert without clinical signs, some young naively exposed horses may show visible signs of nasal discharge and coughing (Allen, 2002; Allen et al., 2004). Previously exposed horses have immune memory that helps reduce the clinical severity of the disease and are infected for only a short duration (Kydd et al., 1994a, b; Allen, 2002). Based on the age and the level of immunity in the infected horse, the respiratory infection may be mild in older horses, pregnant mares, and previously exposed horses, even following virus reactivation from latency (Allen et al., 2004). Experimental infection using the virulent Ab4 strain of EHV-1 revealed a biphasic pattern of pyrexia which may last for up to 10 days (Gibson et al., 1992a,b,c). The clinical picture of the disease includes moderate depression and anorexia, conjunctivitis and serous ocular discharge, and notably a serous nasal discharge which rapidly progresses to mucoid and mucopurulent discharge (Allen et al., 2004). The presence of mucopurulent discharge can be associated with a secondary bacterial infection which may exacerbate the disease. There is progressive lymphadenopathy mainly affecting submandibular lymph nodes (LN) (Allen et al., 2004) and evidence of leukopenia (both lymphopenia and neutropenia) have also been reported (Doll et al., 1954b; Lunn et al., 1991; McCulloch et al., 1993). Occasionally, retropharyngeal LN may also be enlarged and become palpable for some days (Allen et al., 2004). LN may reach maximum size between 3 to 10 days and may remain enlarged for several weeks following infection (Allen et al., 2004). In some infected foals, EHV-1 may reach the lungs inducing bronchopneumonia as a result (Crabb and Studdert, 1996).

While infected horses may occasionally cough, the severity and duration of clinical signs of the disease in a horse are influenced by proper hygiene and rest from exercise or training (Mumford and Rossdale, 1980). Generally, the upper respiratory tract disease (URTD) associated with EHV-1 infection is short-lived and of acute course with clinical symptoms and nasal shedding of the virus manifesting for the first few days following infection (Allen, 2002). Although the prognosis of URTD from EHV-1 is good with spontaneous recovery by the end of the second week of onset of infection, severe bacterial co/secondary infection can prolong the disease and undermine the prognostic chances of survival (Allen, 2002). Upon recovery from URTD caused by EHV-1, some horses may develop non-specific bronchial hypersensitivity, resembling chronic obstructive pulmonary disease, which may hinder their performance and lead to poor performance syndrome (Mumford and Rossdale, 1980).

### Abortion, Neonatal and Perinatal Disease

The associated health implications of EHV-1 extend beyond causing URTD since the virus may invade other organs causing more pronounced disease manifestations (Allen, 2002). One of the sequelae of EHV-1 URTD is abortion in which the virus travels to distant sites such as the reproductive tract by cell-associated viremia or latent viral reactivation (Allen et al., 1999) thereby inducing premature detachment of the fetus from the placenta, stillbirth, or weak neonatal foals (Reed and Toribio, 2004). Pregnant mares infected with the virus may abort spontaneously without prior signs of primary URTD by EHV-1 (Dimock, 1940; Dimock et al., 1942; Bryans and Allen, 1986; Smith K. et al., 1992; Mumford et al., 1994). The important roles exerted by host immune and inflammatory responses, and vascular coagulation cascades mediating EHV-1-induced abortion have not been fully elucidated (Allen et al., 2004). However, infection of the endothelium of a pregnant uterus by EHV-1 results in vasculitis particularly affecting the small vascular networks of the glandular endothelia of microcotyledons (Jackson et al., 1977; Edington et al., 1991; Smith K. et al., 1992; Smith et al., 1993). Within 9–13 days post-infection, endothelial cell infection becomes widespread resulting in multifocal vasculitis of the affected blood vessels (Allen et al., 2004). The appearance of microthrombosis within blood vessels may sometimes promote thrombo-ischemic necrosis of the cotyledons and intercotyledonary stroma causing the fetus to detach from the placenta (Smith et al., 1993). The aborted fetus dies of anoxia following a rapidly progressive separation of the placenta-endometrium immediately before fetal expulsion (Allen, 2002). Widespread vascular endothelial damage may cause the fetus to be aborted even before any detectable level of virus is transferred via the placenta to the fetus (Smith K. et al., 1992).

Experimentally induced abortions by EHV-1 in which virus was not recovered from the aborted fetus have been reported to be as a result of either maternal stress or pyrexia (Gleeson and Coggins, 1980; Carrigan et al., 1991). In another experimental study, the extent of uterine vasculitis and intercotyledonary necrosis corresponds to reduced viral burden in the aborted fetus with fewer lesions found in mares aborting virus-positive fetuses (Smith et al., 1993, 1997). The severity of disease leading to abortion is usually dependent on certain factors including the virulence of the EHV-1 strain involved, the level and magnitude of viremia, and the hormonal state of the pregnant mare. More virulent strains of EHV-1 such as Ab4 have been reported to produce more pathologies including abortion at a higher rate in pregnant mares than the less virulent strains like V592 (Mumford et al., 1994). The pathogenesis of EHV-1-induced abortion by the less virulent strains of EHV-1 is not clear but it appears that those strains have reduced affinity for endothelial cell invasion (Allen et al., 2004). It has also been reported that the magnitude rather than the duration of viremia is a significant correlate of abortion induced by EHV-1 during an experimental challenge (Mumford et al., 1994). Similarly, hormones such as prostaglandin and chorionic gonadotrophin (CG) released by the placenta have been reported to exert some roles in reactivating the virus and initiating abortion (Allen et al., 2004; Paillot et al., 2008). EHV-1 may be transferred by the placenta to the fetus thereby inducing multi-organ pathologies. EHV-1-infected fetuses that are born alive become sick either at conception or within 1–2 days of birth (Bryans et al., 1977; Dixon et al., 1978; Hartley and Dixon, 1979; McCartan et al., 1995). However, such foals rapidly deteriorate and soon die. Infected foals develop severe respiratory distress, which increases the risk of viral pneumonia or bacterial co/secondary infection, leading to respiratory failure within a few days (Crabb and Studdert, 1996; Allen et al., 1999). Foals infected with EHV-1 can also show signs of gastrointestinal disorder (manifested in diarrheic excretion) and neurological deficiencies such as visual and vestibular defects (Dixon et al., 1978). Prognosis is grave and no treatment is available to stop the fatal clinical deterioration of health in infected foals. It has been suggested that congenital defects resulting from EHV-1 infection may be epizootic, especially during sporadic outbreaks of EHV-1-induced abortion (Allen, 2002).

### Myeloencephalopathy

Another clinical sequel of EHV-1 respiratory disease is the neurological form of the disease termed EHM, sometimes appearing after 1 week of infection (Crabb and Studdert, 1996; Donaldson and Sweeney, 1997; Wilson, 1997; Pusterla et al., 2009). Neurologic symptoms may be simultaneously present with or without respiratory disease or abortion (Timoney, 1992; Hahn et al., 1999). Fundamentally important in the spread of EHV-1 is cell-associated viremia which effectively disseminates the virus to the vascular network of the CNS. The intracellular localization of EHV-1 during infection protects it from the neutralizing effect of circulating antibody, enabling an efficient spread of the virus throughout the body, including to the CNS, even when high levels of antibodies are present (Bryans, 1969). Like other members of *Herpesviridae*, EHV-1 can be transmitted directly from cell to cell independent of an extracellular phase (Allen, 1986). The vascular endothelium of the CNS serves as the preferred site for EHV-1 replication following the transfer of the virus from circulating mononuclear cells (Wilson, 1997). Endothelial cell invasion and the accompanying inflammation of the vasculature of the CNS is central to the neurological syndrome caused by EHV-1 (Jackson and Kendrick, 1971; Jackson et al., 1977; Patel et al., 1982; Edington et al., 1986). The vasculitis of the endothelium resulting from EHV-1 infection may be as a result of two different events; first, direct damage of the vascular endothelium during EHV-1 replication, and second, immune complex formation between EHV-1 and antibody (Arthus-type reaction) (Reed and Toribio, 2004). Common hallmarks of the neurological form of EHV-1 are the development of vasculitis with or without hemorrhage, and thrombo-ischemic necrosis of the microvasculature of the brain or CNS (Allen et al., 2004). The observed clinical signs in infected horses are a culmination of the vasculitis, edema, hemorrhage, ischemia, and necrosis resulting from the viral predilection for vascular endothelium (Reed and Toribio, 2004). Indeed, the ability of certain EHV-1 strains to inflict damage on the CNS is not reflective of their neurotropic trait but instead an endotheliotropic attribute (Jackson and Kendrick, 1971; Jackson et al., 1977; Edington et al., 1986, 1991; Nowotny et al., 1987). However, the observation of neural lesions and chorioretinopathy during experimental infection of specific pathogen-free ponies indicates that at least some EHV-1 strains may be neurotropic (Slater et al., 1992). There seems to be no satisfactory scientific explanation for the variable incidence of EHM and different clinical manifestations observed during outbreaks of EHV-1 (Whitwell and Blunden, 1992; McCartan et al., 1995). Several factors including sex, age, immune status of the horse, the reproductive status of the mare (including the stage of gestation), the severity of infection, the type of strain, and perhaps the route of transmission determine the clinical picture of EHV-1 infection (Studdert et al., 1984; Whitwell and Blunden, 1992; McCartan et al., 1995).

Clinical signs of the neurologic disease may become apparent within 2 weeks of URTD or may occur without any antecedent sign of the disease (Wilson, 1997). The clinical presentations are highly variable and widespread depending on the site of neurologic impact and usually peak between 2 and 3 days of onset (Allen et al., 2004). Generally, there is anorexia, pyrexia, edema of the distal limb, abortion, fetal death, or neurologic syndrome, and these are variable in different horses (McCartan et al., 1995). The extent of neurological dysfunction ranges from temporary ataxia with an abnormal gait to complete paralysis. Conscious proprioceptive deficits have also been observed (Allen et al., 2004). The neurological disorders affect mainly the hind limbs, although complete recumbency or tetraplegia have also been observed (Kohn and Fenner, 1987; McCartan et al., 1995; Allen et al., 2004). In some cases, there are signs of bladder dysfunction with accompanying urinary incontinence and scalding of the perineal area or urinary retention which may lead to colic (Franklin et al., 1985). Horses that are non-recumbent have a good prognosis unlike recumbent horses that may suffer additional complications such as pneumonia, colic or bladder rupture (George, 1990; McCartan et al., 1995; Allen et al., 2004) and are generally euthanized.

#### Recent Outbreaks of EHM

Outbreaks of disease resembling EHM, among domestic horse populations, have been recorded for centuries. Today, a resurgence in the number of EHM cases across the world has necessitated the classification of this syndrome as an emerging disease of the horse. According to the Center for Emerging Issues report of 2007, EHM satisfies the requirement for an emerging viral disease premised on (1) the more virulent nature of the circulating EHV-1 strains than previously reported and (2) increased incidence of the disease with a heightened case fatality rate (APHIS and USDA, 2008). Increased outbreaks of EHM were reported not only in North America and Europe, but also in Africa, Oceania, and Asia (Henninger et al., 2007; Gryspeerdt et al., 2011; Tsujimura et al., 2011; Burgess et al., 2012; Pronost et al., 2012; Traub-Dargatz et al., 2013; Walter et al., 2013; Estell et al., 2015; van Galen et al., 2015; McFadden et al., 2016; Negussie et al., 2017). The recent increased incidence of EHM during EHV-1 outbreaks supports the observation that the currently circulating neuropathogenic EHV-1 strain has evolved into a more pathogenic strain producing a higher rate of morbidity and mortality than previously (APHIS and USDA, 2007). EHM has been associated with an A_2254_→G_2254_ mutation in ORF 30 encoding the DNA polymerase of EHV-1. Generally, non-neuropathogenic strains possess asparagine at position 752 of DNA polymerase which is substituted by aspartic acid in neuropathogenic strains (Nugent et al., 2006; Goodman et al., 2007). This relationship is strong but not always true in field outbreaks, and other factors may contribute to neuropathogenicity (Lunn et al., 2009; Pronost et al., 2010). Approximately, 14 percent to 24 percent of EHV-1 strains from horses displaying clinical signs of EHM lack this genetic indicator suggesting that the so-called non-neuropathogenic genotype of EHV-1 can also cause EHM (Nugent et al., 2006; Perkins et al., 2008). This disease condition is a major concern for the horse industry considering its negative impact on the economic health of the industry.

The associated risk factors for this increased incidence of EHM are still poorly defined. However, outbreaks have been reported mostly at places such as racetracks, riding schools, and veterinary hospitals where horses from different origins congregate (Kohn et al., 2006; Henninger et al., 2007; Traub-Dargatz et al., 2013). The high stocking density of stabled horses during events such as horse racing may facilitate the quick spread of EHM by direct contact when outbreaks occur. International movement of horses has also played a role in some recent outbreaks of EHM (APHIS and USDA, 2007; Barbić et al., 2012). Other factors that have been reported to facilitate increased incidence of EHM include poor biosecurity measures and presence of stressors (Traub-Dargatz et al., 2013; Pusterla and Hussey, 2014) along with other ill-defined environmental and host factors (Perkins et al., 2009). Importantly, the mutant EHV-1 (G_2254_) is now widely distributed within horse populations which implies a tendency toward the increased incidence and severity of recent EHM outbreaks.

### Ocular Disease

Occasionally and particularly in foals, respiratory tract infection with highly pathogenic EHV-1 strains is associated with severe ocular lesions such as chorioretinitis or uveitis (Del Piero and Wilkins, 2001). Within 3–5 weeks of URTD by EHV-1, foals may develop three distinct types of chorioretinal lesions (focal, multifocal or diffuse) without uveitis (Slater et al., 1992). Although the first report of EHV-1 associated chorioretinitis was in Ilamas and alpacas (Rebhun et al., 1988; House et al., 1991), this condition has also been documented in natural outbreaks of paralytic EHV-1 infection involving a mare and foal (Whitwell and Blunden, 1992). More recently, an incidence rate of 50–90% of horses was shown to develop chorioretinal lesions during an experimental challenge with EHV-1 (Hussey et al., 2013). Like the pathogenesis of EHV-1 induced abortion and neurologic syndromes, replication of EHV-1 in the vasculature of the chorioretina may result in ischemic necrosis resulting in visual impairment (Slater et al., 1995). Apart from chorioretinitis, uveitis is another ocular condition seen in some foals following outbreaks of EHM in mares and stallions (McCartan et al., 1995). Young foals that come in close contact with EHM-infected mares and stallions are at high risk of developing ocular disease associated with EHV-1 (McCartan et al., 1995).

## Ehv-1 Interactions With the Host Immune System

Several efforts over the decades have been channeled toward understanding and characterizing the protective host immune response against EHV-1 which could be exploited to advance diagnostic approaches and vaccine development. However, despite over 80 years since EHV-1 was first recognized, we do not yet fully understand how the virus’ interaction with the host immune response can be harnessed for the development of more effective immunotherapy.

Following experimental infection of horses with a virulent strain of EHV-1, viral components were immediately detected in the regional LN of the respiratory tract within 12 hpi (Kydd et al., 1994a, b). This indicates that the virus has close interaction with the host immune system during early infection, and triggers an immediate host response consisting predominantly of inflammatory cytokines. Johnstone et al. (2016) recently reported the upregulation of pro-inflammatory cytokines in an equine endothelial cell (EEC) model at 10 hpi with either neurovirulent or non-neurovirulent strain of EHV-1. Similarly, studies from our laboratory have shown the ability of EHV-1 to produce type-I IFN induction in EECs as early as 3 hpi which is subsequently followed by a progressive decline by 6 and 12 hpi (Sarkar et al., 2015, 2016a,b; Oladunni et al., 2018, 2019). The initial upregulation of inflammatory cytokines during early EHV-1 infection helps to activate the adaptive arm of the host immune response toward eliminating the viral antigen. However, there is a concern that this response may be a double-edged sword: induced proinflammatory cytokines and activated coagulative responses that follow may also induce pathology that could negate their antiviral benefits.

It has also been reported that the host humoral immunity toward EHV-1 infection is temporary (van der Meulen et al., 2006) making horses susceptible to re-infection even after vaccination. EHV-1 infected horses usually display virus-neutralizing (VN) and complement-fixing (CF) antibodies within 2 weeks of infection (Hannant et al., 1993). This has proved important for early diagnosis of EHV-1 which is critical to quickly forestall the spread of infection to naïve, unexposed horses. While VN antibodies are type-specific and give longer protection (up to a year), there is cross-reactivity between CF antibodies of EHV-1 and EHV-4; however, CF antibodies only last for about 3 months. The host humoral immunity is usually targeted to recognize epitopes on the surface of envelope glycoproteins of EHV-1 (Allen and Yeargan, 1987; Crabb et al., 1991; Packiarajah et al., 1998; Perkins et al., 2019), and IgGa, IgGb, IgGc, IgGd, IgM, and IgG(T) antibody isotypes have all been detected in EHV-1-infected horses (Paillot et al., 2008; Wagner et al., 2015; Perkins et al., 2019). The protective effect of circulating antibodies during infection with EHV-1 is limited once a state of cell-associated viremia is established. However, local protection from freely circulating EHV-1 in the URT can be achieved with the help of mucosal antibody, particularly the IgA isotype. As a result, VN antibodies are helpful during the initial respiratory infection produced by EHV-1 but incompetent to provide protective immunity against the more severe sequelae: abortion and neurologic diseases which are enabled by the establishment of cell-associated viremia.

As with other intracellular pathogens, successful elimination of EHV-1 following the establishment of cell-associated viremia is dependent on an activated cytotoxic T lymphocyte (CTL) response. An upregulated CTL response has been reported following experimental EHV-1 infection (Breathnach et al., 2006) with IFN-γ playing a critical role in activating antigen-presenting cells and enhancing the antiviral effects of the circulating cytotoxic CD8 T cells. There is a relationship between the level/frequency of circulating CTL and protection from clinical signs of the diseases caused by EHV-1. Adult ponies with a previous history of exposure to EHV-1 produce high levels of EHV-1 specific circulating CTL and show fewer signs of clinical disease when compared to young ponies with low EHV-1 specific circulating CTL (O’Neill et al., 1999). This highlights the important role played by CTL precursor or memory cells during EHV-1 re-infection which may prove useful for testing the efficiency of EHV-1 vaccines in horses. The gene product of EHV-1 IEP encoded by ORF 64 has been reported to have epitopes specifically targeted by CTL from horses expressing the MHC class 1 A3/B2 serological haplotype (Soboll et al., 2003; Kydd et al., 2006). While this finding looks exciting for the development of vaccines for the immunodominant 95% population of Thoroughbred horses that express these serological haplotypes, further research is warranted to identify EHV-1 gene products expressed by other MHC class I haplotypes present in outbred horse populations.

## Laboratory Diagnosis

A rapid diagnosis of URTD associated with EHV-1 within a group of horses is highly desirable to aid therapeutic decisions and shape future control strategies to prevent an epidemic outbreak of the disease (Allen, 2002). Usually, the presenting clinical sign alone is not sufficient to reach a precise diagnosis as the initial clinical presentation may also resemble that of equine influenza, adenovirus, etc. As a result, laboratory diagnostic confirmation of EHV-1 induced URTD is predicated on the ability to detect the virus in submitted clinical materials. Polymerase chain reaction (PCR) is a useful diagnostic tool for an immediate identification/detection of genomic materials of EHV-1 in submitted clinical or pathological samples such as aborted fetus, placenta, nasal swabs, nasal discharges, brain and spinal cord, paraffin-embedded archival tissues, and infected cell cultures (Ballagi-Pordany et al., 1990; Borchers and Slater, 1993; Kirisawa et al., 1993; Lawrence et al., 1994; Mackie et al., 1996). Perhaps the most sensitive diagnostic tool for EHM is the real-time PCR which can discriminate isolates possessing the single point mutation in the ORF30 gene associated with the neurologic phenotype of the disease (Smith et al., 2012). Evaluation of this newly improved real-time PCR revealed that it is more specific, besides being capable of discriminating an A_2254_ from a G_2254_, than an old assay (Allen, 2007) that produces false dual positive results in detecting viral components in clinical samples. Allelic discrimination of viral nucleic acid during field outbreaks of EHV-1 is a useful epidemiological tool that allows for a rapid diagnosis and timely imposition of appropriate control measures required to prevent viral spread. It does not, of course, definitely diagnose EHM as the genetic correlation is not absolute as discussed above. One major caveat to the use of PCR is that it is not able to distinguish nucleic acid from a viable virus from that of a non-viable virus. It has been reported that the agreement between PCR and virus isolation is about 85–90 percent (Allen et al., 2004). This may be a particular concern when interpreting the presence of extremely low levels of viral DNA in clinical samples.

Direct detection of viral antigen from clinical samples using immunofluorescence staining also provides for rapid diagnosis of EHV-1. Detection of viral antigen in impression smears from nasopharyngeal swabs following fluorescent antibody staining can be used to demonstrate a positive EHV-1 infection (Allen, 2002; Allen et al., 2004). Immunohistochemically, EHV-1 antigens can be detected in paraffin-embedded tissues from infected horses using immunoperoxidase staining (Allen et al., 2004). Histopathological examination of paraffin-embedded tissue sections can also be employed to identify pathognomonic lesions typical of EHV-1 infections. However, these findings should also be verified by virus isolation from submitted clinical specimens.

Differential diagnosis of EHV-1 from cases of EHV-4 infection is difficult by serology. Using type-specific antigen, serological diagnostic tests such as enzyme-linked immunosorbent assay (ELISA), complement fixation (CF), and virus neutralization (VN) can be employed on paired serum samples to differentiate between EHV-1 and EHV-4. Serum samples that were taken during the acute and convalescent phases of infection can be screened to determine their titer; i.e., the highest dilution of sera with detectable binding/neutralization for either EHV-1/EHV-4. A type-specific ELISA assay, which detects distinct humoral response to a unique EHV-1/EHV-4 peptide antigen, has been developed and shown to be a valuable tool for a more accurate diagnosis (Lang et al., 2013).

The gold standard technique for a definitive diagnosis of EHV-1 is virus inoculation of cell cultures for isolation of the virus. EHV-1 can be isolated from various cell lines including those from the horse (EEC), rabbit (RK-13), monkey (Vero), and cattle (MDBK) (Allen et al., 2004; Rybachuk, 2009). The cytopathic effect (CPE), develops rapidly in cell cultures as clusters of rapidly enlarging, rounded, and detached cells which are characteristically herpetic in appearance (Allen et al., 2004). The application of a quick diagnostic technique such as PCR in conjunction with virus culture and isolation is useful to characterize the virus for further epidemiological investigation. Confirmatory diagnosis of EHV-1 should rule out other differentials such as equine arteritis virus, influenza virus, adenovirus, rhinovirus, EHV-4, and *Sarcocystis neurona* infection, which may all present disease phenotypes that mimic EHV-1 infection.

## Current Treatment and Control Recommendations

There is no specific drug effective against EHV-1 disease conditions. However, good hygiene and management practices together with symptomatic treatment of infected horses may help curtail the spread of the viral infection. The current recommendations for treatment of recumbent horses include offering supportive care, nutritional care and rehydration, frequent bladder and rectal evacuation to prevent colic, and reduction of CNS inflammation (Wilson and Erickson, 1991; Goehring and Lunn, 2008). Symptomatic treatment with non-steroidal anti-inflammatory agents as an adjunct therapy may be helpful (Reed and Toribio, 2004; Lunn et al., 2009). Similarly, corticosteroids and immunomodulatory agents may be used to symptomatically treat early signs in cases of EHM. However, there is no evidence-based study to support the effectiveness of either drug class and caution must be applied not to reactivate virus shedding in latently infected horses (Lunn et al., 2009; Rybachuk, 2009). Corticosteroids are suggested to be protective against the cellular response to CNS infection thereby preventing the development of hemorrhage, edema, vasculitis, and thrombosis that are common early signs of EHM, and their use should be reserved for severe cases of EHM (Lunn et al., 2009).

Similarly, the administration of immunostimulants before horses are exposed to stressors could help prevent viral reactivation and replication but their value for treating EHV-1 infection is yet to be ascertained (Lunn et al., 2009). Antiviral drugs, especially virustatic agents like acyclovir derivatives, are theoretically beneficial for EHV-1 infection (Garré et al., 2007). Beside acyclovir, prophylactic administration of valacyclovir hydrochloride has been tried in experimentally infected horses with demonstrable benefits (Maxwell et al., 2017). Ganciclovir has been demonstrated to be the most potent inhibitor of EHV-1 infection in an *in vitro* study that investigated the efficacy of many antivirals against EHV-1 (Garré et al., 2007), and in a more recent study, it also offers a much-improved bioavailability, *in vivo* (Carmichael et al., 2013) compared to acyclovir.

Like other herpesviruses, EHV-1 infection is more complicated than most other viral infections; the establishment of persistent latent infection ensures that EHV-1 is naturally maintained in horse populations all year-round. Also, EHV-1 has evolved a vast array of strategies to avoid many components of the host innate and adaptive immune responses (van der Meulen et al., 2006). As a result, an efficient EHV-1 vaccine must be able to invoke strong and sustained levels of humoral and cell-mediated immunity against the virus. In addition, since the establishment of cell-associated viremia is required for the development of abortion as well as EHM, an effective vaccine candidate must, also, be able to stimulate those immune responses needed to block the development of cell-associated viremia. The currently available commercial vaccines against EHV-1 in North America are in the form of inactivated whole virus vaccine and modified live vaccine (MLV). In a recent study, three groups of horses were either administered with a saline placebo or vaccinated with Rhinomune (Boehringer Ingelheim), derived from *Rac*-*H* strain (Mayr et al., 1968), or Pneumabort K-1B (Zoetis), which contains EHV-1 1P and 1B strains (Allen et al., 1985). The efficacy of either vaccine was then evaluated following an EHV-1 challenge experiment with the Findlay OH03 strain. Observed clinical signs of disease following infection with EHV-1 in the saline control group included pyrexia, depression, anorexia, coughing, nasal discharge, and dyspnea. Both the Rhinomune (Boehringer Ingelheim), an MLV, and Pneumabort K-1B (Zoetis), an inactivated vaccine, reduced the clinical incidence of disease with the former offering better protection (Goehring et al., 2010). However, the effectiveness of either vaccine in preventing EHV-1 induced abortion or EHM is still far from proven. EHV-1 antigen is also incorporated in some multivalent vaccines marketed across the US in their inactivated forms. Recombinant vaccine models expressing EHV-1 gB, gC, and gD reduced the initial nasal viral shedding in vaccinates but offered less protection against cell-associated viremia and clinical signs of disease (Minke et al., 2006; Soboll et al., 2006). Intriguingly, a recombinant vaccine expressing an EHV-1 IE gene, encoded by ORF 64, significantly reduced cell-associated viremia in vaccinated ponies, however, its effect on EHV-1 induced abortion and EHM remain inconclusive (Soboll et al., 2010). There is currently no available vaccine that completely prevents EHV-1 infection, or EHV-1-induced cell-associated viremia or latency, and EHV-1 myeloencephalopathy has been reported in vaccinated horses (Kohn and Fenner, 1987; Wilson and Erickson, 1991; Friday et al., 2000; Henninger et al., 2007). Nonetheless, it is recommended to vaccinate every horse that is at risk of exposure to EHV-1 to help reduce the severity of EHV-1-related clinical manifestations. The updated version of American Association of Equine Practitioners (AAEP) guidelines for vaccination of adult horses provides a detailed recommendation for vaccinating against EHV-1 (Anonymous, 2015).

Control measures for curtailing EHV-1 infection are aimed at reducing viral dissemination to susceptible horses and also at preventing the reactivation of the virus in latently infected horses (Allen, 1986; Ostlund et al., 1990; Ostlund, 1993). Infected sick horses are primary sources of infectious EHV-1, and as such, should be quarantined to prevent direct contact with un-infected horses. Also, infected materials such as aborted fetus or uterine contents, including placenta, should be disposed of appropriately to curtail the spread of EHV-1 (Reed and Toribio, 2004). High-level biosecurity measures should be put in place in farms and all visitors should be encouraged to use a footbath and wash their hands before entering or leaving horse farms. Infected equipment must be disinfected and disposed of, and different equipment and workers must operate on affected and unaffected horses to prevent horizontal transmission of the disease (Allen, 1986). Movement of horses and visitors should be restricted onto and off the infected farm premises until laboratory tests indicate negative results for EHV-1 infection. Newly acquired horses should be quarantined from the rest of the herd for at least 3 weeks and must be certified negative for EHV-1 before being allowed to join the resident population. Horse owners and horse farmers should immediately report EHM outbreaks to relevant government agencies to contain the spread of the disease and to help formulate policies against future outbreaks.

## Concluding Remarks

EHV-1 is still persistent among domesticated horses around the world and the vaccines currently available are not completely protective, especially against EHM. As a result, EHV-1 still poses a huge threat to the horse industry and efforts geared toward preventing the outbreak of the disease are strongly encouraged. Detailed information on both the host and certain environmental factors that enable the recent incidence of EHV-1 myeloencephalopathy are still elusive. Further research is required to determine robust epidemiological factors that promote the disease. Most research on how EHV-1 modulates host immune responses have been carried out *in vitro* with only a few studies investigating the immunomodulatory effects of EHV-1 *in vivo*. More *in vivo* studies focusing on viral properties that are important for the evasion of host immunity will help to expose putative therapeutic targets of EHV-1. Future progress in treatment and control of EHV-1 hinges on the combined application of detailed epidemiological data and in-depth knowledge of how the sophisticated viral biology promotes pathogenesis.

## Author Contributions

FO wrote the manuscript while both DH and TC edited it.

## Conflict of Interest

The authors declare that the research was conducted in the absence of any commercial or financial relationships that could be construed as a potential conflict of interest.
